# Influence of Novel “Umbrella”-Type Ladle Shroud on Liquid Steel Flow in a Two-Strand Slab Tundish: Physical and Numerical Modelling

**DOI:** 10.3390/ma19010096

**Published:** 2025-12-26

**Authors:** Adam Cwudziński, Lukáš Fogaraš, Jaroslav Demeter, Peter Demeter, Branislav Buľko

**Affiliations:** 1 Department of Metallurgy and Metals Technology, Faculty of Production Engineering and Materials Technology, Czestochowa University of Technology, Armii Krajowej 19 Ave, 42-201 Czestochowa, Poland; 2 Institute of Metallurgical Technologies and Digital Transformation, Faculty of Materials, Metallurgy and Recycling, Technical University of Košice, Letná 1/9, 042 00 Košice, Slovakia; lukas.fogaras@tuke.sk (L.F.); jaroslav.demeter@tuke.sk (J.D.); peter.demeter@tuke.sk (P.D.); branislav.bulko@tuke.sk (B.B.)

**Keywords:** tundish, ladle shroud, hydrodynamics, slab casting, numerical simulations, water modelling

## Abstract

In this paper, the influence of the novel design of a ladle shroud (LS) on the liquid steel flow structure inside the working volume of a two-strand slab tundish was assessed, determining the best solutions for LS use to achieve the optimal level of active flow zones and protect the tundish lining. A 0.33 scale water model was used for physical experiments. Numerical simulations were carried out in the Ansys-Fluent 12.1 software for a 1:1 scale tundish. The effect of the influence of LS type, LS immersion depth, LS side ports position, LS misalignment and casting speed was examined. Finally, the use of the “umbrella” ladle shroud allows stable hydrodynamics to be maintained even with shroud misalignment. Moreover, the “umbrella” ladle shroud effectively decreases the average velocity of liquid steel inside the tundish and significantly decreases shear stresses and dynamic pressure at the tundish lining in the tundish pouring area.

## 1. Introduction

The past quarter of a century of the 21st century has seen rapid development in continuous steel casting technology [1,2,3,4,5,6,7,8,9,10]. In the technical and technological field, both universal solutions and solutions dedicated to selected steelworks have been developed. The mechanisms of mass, momentum and heat transport are directly correlated with the hydrodynamic state of metallurgical reactors, which provides the basis for the search for unique solutions and measurable benefits in continuous casting under industrial conditions. One of the key elements of continuous steel casting equipment is the tundish. The tundish, filled with liquid steel, ensures an even supply to individual moulds and flexible modification of casting conditions for individual strands in the secondary cooling zone [11,12,13,14]. Since tundishes have a varied working space design, as flow reactors, they require analysis in each case to assess the hydrodynamic system forming in their working space [15,16,17,18,19,20].

Depending on the number of outlets and their position relative to the feed zone, the momentum of the feed stream determines the intensity of the inertial forces that shape the recirculation zones in the working volume of the tundish. In the case of classic design solutions, the supply stream can already be influenced in the pouring zone by equipping the tundish with a subflux turbulence controller [21,22,23,24]. Moving away from the pouring zone, the movement of liquid steel is modified by dams or weirs in order to stimulate upward movement towards the free surface of the liquid steel. The upward movement facilitates the removal of non-metallic inclusions and supports the liquid steel refining process [25,26,27,28]. In this respect, solutions based on argon injection through gas-permeable barriers are equally effective [29,30,31,32]. In the context of activating and controlling recirculation areas, tundishes are equipped with electromagnetic stirrers or channel induction heating [33,34,35,36]. In addition, the design of the ladle shroud used to feed the tundish is being intensively developed. Ladle shrouds not only protect the liquid steel stream from re-oxidation or excessive cooling, but also effectively reduce the driving force of the feed stream, controlling the cleanness of the liquid steel. In addition, ladle shrouds with a modified working space effectively influence the hydrodynamic structure in the working space of the tundish [37,38,39,40,41,42,43,44].

Finally, analysis of the tundish metallurgy field shows that many interesting solutions are being proposed for two-strand tundishes; hence, the evolution of slab continuous casting technology is continuous [45,46,47,48,49].

In this paper, the influence of the novel design of a ladle shroud (LS) on the liquid steel flow structure inside the working volume of a two-strand slab tundish was assessed, determining the best solutions for LS use to achieve the optimal level of active flow zones and protect the tundish lining.

## 2. Tundish and Ladle Shroud Description

**Figure 1:**
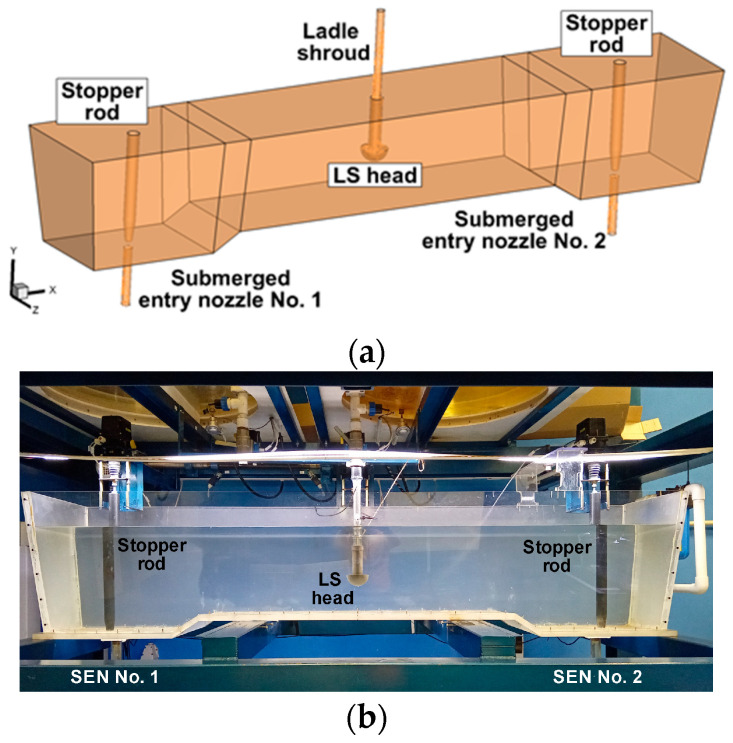
Sixty-five-ton ladle: (**a**) full-scale tundish numerical model, (**b**) 0.33-scale tundish physical model.

The tested object was a tundish designed for casting slabs (Figure 1a). The capacity of the tundish is 65 tons of liquid steel. The dimensions of the tundish are described in detail in [50]. The tundish is equipped with an “umbrella” ladle shroud (ULS) with an internal diameter of 0.0848 m [51]. The new ladle shroud is terminated with a dome with an external radius of 0.139 m. The feed stream to the tundish is distributed to the working volume of the dome through two rectangular side ports with dimensions of 0.06 m × 0.105 m. The liquid steel flows out of the tundish through submerged entry nozzles with an internal diameter of 0.09 m. The flow rate of molten steel to the moulds is regulated by a stopper rod system. The bottom of the tundish outlets zone is lowered relative to the bottom of the feed zone in order to limit the transfer of slag phase to the liquid steel in the final stage of the casting sequence and the contamination of the slabs. Figure 1b shows a physical model of the tundish on a scale of 0.33. The capacity of the tundish physical model is 0.4 tons of water.

The physical model of the tundish is part of a laboratory stand equipped with two ladles and two moulds for casting slabs. The stand is equipped with an automatic water flow control system directly correlated with the casting speed, adjusting the filling speed of the moulds depending on the water level in the tundish. Table 1 presents the technical and technological variants of the casting sequence analysed during the laboratory trials. The trials concerned the casting of two slabs with dimensions of 1.15 m × 0.225 m at speeds of 1 m/min and 1.6 m/min. Two immersion depths of the proposed ladle shroud were tested, i.e., 0.36 m and 0.6725 m. During the tests, two positions of the side ports in the ladle shroud were also tested. In the first variant, the side ports were directed towards the stopper rods (ULS-O), while in the second variant, they were directed towards the side longitudinal walls of the tundish (ULS-W). In addition, the effect of a three-degree deviation of the ladle shroud from the axis towards stopper rod No. 2 on the hydrodynamic structure was verified. The results obtained for casting with a modified ladle shroud were compared to the casting variant with a standard ladle shroud (straight pipe with a constant internal diameter of 0.09 m).

## 3. Methodology

The first stage of the research path was the water model physical trials, during which the residence time distribution curves were monitored for different casting conditions. Next, for the most acceptable variants of modern ladle shroud use, additional numerical simulations were performed to detect the best hydrodynamic conditions inside the liquid steel bulk.

The basic similarity criterion used in the physical experiments was Froude’s criterion. Froude’s criterion estimates the relation between momentum forces and gravitational forces in the considered high-temperature system. Water was used for modelling the liquid steel’s behaviour because kinematic viscosity is nearly the same for both fluids [52,53].

(1) $${\left(\frac{u^{2}}{gL}\right)}_{m}={\left(\frac{u^{2}}{gL}\right)}_{p}$$

(2) $$Q_{m}=\lambda^{2.5}Q_{ls}$$

where *L*—characteristic length (m); *u*—velocity of fluid (m/s); *g*—acceleration of gravity (m/s^2^); *Q_m_*—volumetric flow rate of water in the physical model (m^3^/s); *Q_ls_*—volumetric flow rate of liquid steel in the industry tundish (m^3^/s); *λ*—scale factor.

Numerical simulations were carried out in the Ansys-Fluent 12.1 software (ANSYS, Inc., Canonsburg, PA, USA) for a 1:1 scale tundish and the behaviour of liquid steel. The isothermal numerical model includes the mass and momentum phenomena of fluid described below.

(3) $$\frac{D\rho }{Dt}=-\rho \left(\nabla \cdot u\right)$$

(4) $$\rho \frac{Du}{Dt}=\mu \nabla^{2}u-\nabla p+\rho g$$

where *t*—time (s); *μ*—viscosity of fluid (Pa·s); *p*—pressure (Pa).

The turbulent nature of the system was described via a realizable *k*-*ε* approach [54]. The basic equations in this model are written as:

(5) $$\frac{𝜕\rho k}{𝜕t}+\frac{𝜕\rho u_{i}k}{𝜕x_{i}}=\frac{𝜕}{𝜕x_{i}}\left(\frac{\mu }{\sigma_{k}}\frac{𝜕k}{𝜕x_{i}}\right)+G-\rho \varepsilon$$

(6) $$\frac{𝜕\rho \varepsilon }{𝜕t}+\frac{𝜕\rho u_{i}\varepsilon }{𝜕x_{i}}=\frac{𝜕}{𝜕x_{i}}\left(\frac{\mu }{\sigma_{\varepsilon }}\frac{𝜕\varepsilon }{𝜕x_{i}}\right)+C_{1}-C_{2}\frac{\varepsilon^{2}}{k+\sqrt{\nu \varepsilon }}$$

(7) $$\nu =C_{\mu }\frac{k^{2}}{\varepsilon }$$

(8) $$C_{1}=\max\left\{0.43,\frac{\eta }{5+\eta }\right\}$$

(9) $$C_{\mu }=\frac{1}{A_{0}+A_{S}\frac{kU^{*}}{\varepsilon }}$$

(10) $$A_{s}=\sqrt{6}\cos\varphi$$

(11) $$U^{*}=\sqrt{S_{ij}S_{ij}+\Omega_{ij}\Omega_{ij}}$$

where *t*—time; *u*—fluid velocity; *ρ*—fluid density; *ε*—dissipation rate of kinetic turbulent energy; *k*—turbulent kinetic energy; *G*—generation of turbulence kinetic energy; *μ*—fluid viscosity; *ν*—fluid kinematic viscosity; *S_ij_*—main strain rate tensor; Ω*_ij_*—rotational-rate tensor.

The constants of the model have the following values: *A*_0_ = 4.04, *C*_2_ = 1.9, *σ_k_* = 1.0, *σ_ε_* = 1.2.

Based on the combined model and taking into account the dispersion of plug flow and mass exchange between the zone of active and stagnant flow, the hydrodynamic structure, volume fractions of dispersed plug flow, stagnation and ideal mixing were described [55,56]. Thus, the following equations were used during calculations:

(12) $$C_{\left[-\right]}=\frac{V_{ls}}{W_{m}}\cdot C_{t}$$

(13) θ=tWlsm

(14) $$\frac{V_{s}}{V_{ls}}=1-\frac{Q_{a}}{Q}\cdot \theta_{c}$$

(15) $$\frac{V_{p}}{V_{ls}}=\frac{\theta_{\min}+\theta_{peak}}{2}$$

(16) $$\frac{V_{m}}{V_{ls}}=1-V_{p}-V_{s}$$

where *C*_[–]_—dimensionless concentration of the marker; *V_ls_*—volume of liquid steel in the tundish; *W_m_*—weight of the marker; *C_t_*—temporary concentration of the marker; *θ*—dimensionless time; *t*—time; *W_ls_*—weight of liquid steel in the tundish; *m*—mass flow rate; *V_s_*—volume of stagnant flow in the tundish; *V_p_*—volume of dispersed plug flow in the tundish; *V_m_*—volume of ideal mixing flow in the tundish; *Q*—total volumetric flow rate through the tundish; *Q_a_*—volumetric flow rate through the active region of the tundish; *θ_c_*—dimensionless average mean residence time up to *θ* = 2; *θ_min_*—dimensionless time of the first appearance of the marker; *θ_peak_*—dimensionless time of the maximum concentration of the marker.

The SIMPLEC scheme was used to describe pressure–velocity coupling. Moreover, spatial discretization for gradient and pressure was Green-Gauss Cell-Based and standard, respectively. For pressure, momentum, turbulent kinetic energy and turbulent dissipation rate, second-order upwind discretization was chosen. Under-relaxation factors for solution controls were default, apart from momentum, for which the value amounted to 0.5. The residuals for all equation components were below a value of 10^−5^. The free surface of liquid steel was modelled by wall boundary conditions with zero shear stress. For the tundish lining, ladle shroud and SENs, standard wall function and wall boundary conditions were used. Velocity inlet and outflow boundary conditions were adopted for LS and SENs. Moreover, gravity and full buoyancy effects were included in the numerical model. For the simulation of tracer behaviour, a species model was used. This approach makes it possible to describe the properties of the tracer. Therefore, for maintaining similarity between liquid steel and the tracer, the tracer should have the same properties, especially density, for perfect interaction with liquid steel streams. Hence, the density of the tracer was 7010 kg/m^3^.

Liquid steel flowed into the tundish via ULS at a velocity of 1.52 m/s. The initial kinetic energy of the turbulence was 0.023 m^2^/s^2^, and the dissipation of the kinetic turbulence energy was 0.082 m^2^/s^3^. For faster casting conditions, the velocity at the tundish inlet was 2.43 m/s. In this case, initial kinetic energy and its dissipation increased, respectively, to 0.059 m^2^/s^2^ and 0.337 m^2^/s^3^. The kinetic energy and its dissipation were calculated on the basis of works [57,58]. For the standard ladle shroud, the initial velocity and parameters on turbulence conditions were 1.35 m/s, 0.018 m^2^/s^2^ and 0.054 m^2^/s^3^ for a 1.0 m/min casting speed and 2.16 m/s, 0.046 m^2^/s^2^ and 0.223 m^2^/s^3^ for a 1.6 m/min casting speed. The following physicochemical properties of liquid steel were assumed: density 7010 kg/m^3^ and viscosity 0.007 Pa·s. Numerical simulations and physical experiments were both performed for isothermal conditions.

## 4. Results and Discussions

### 4.1. Physical Trials—Influence of LS Immersion Depth, LS Ports Sides Position and LS Misalignment on Hydrodynamics Inside Tundish

**Figure 2:**
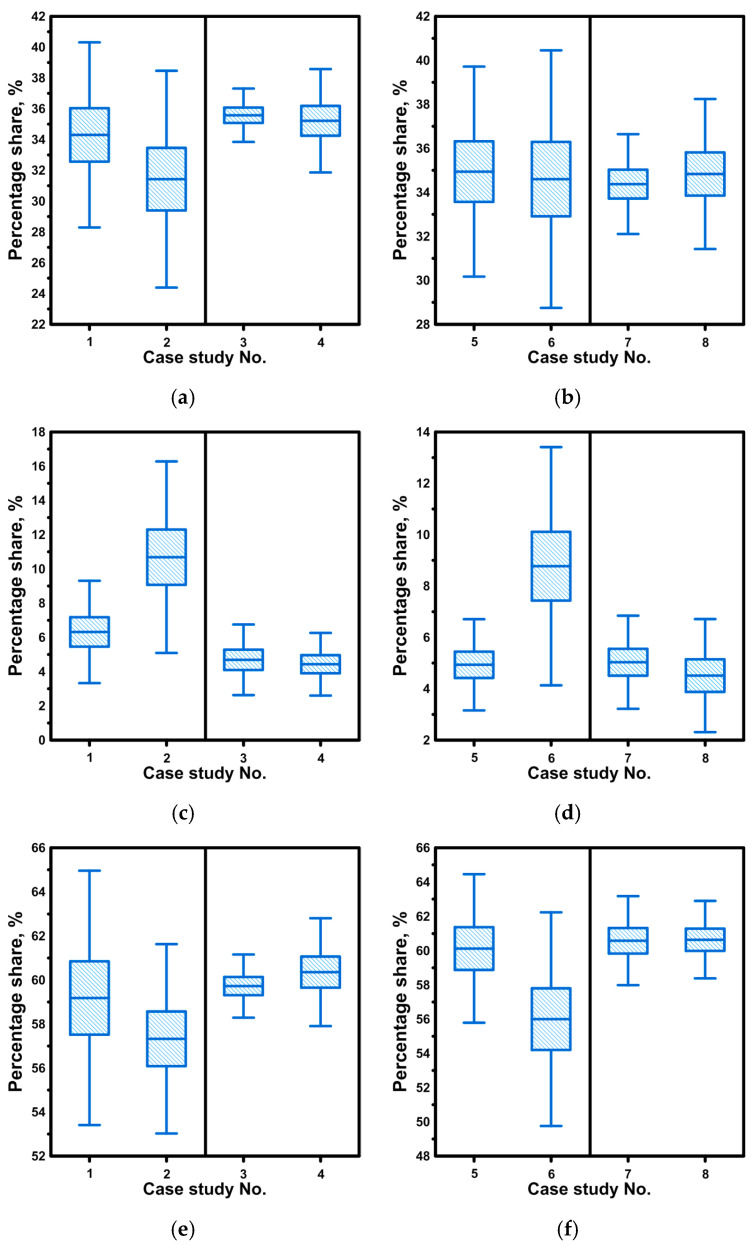
Tundish hydrodynamics: (**a**) stagnant flow zone for LS alignment, (**b**) stagnant flow zone for LS misalignment, (**c**) plug flow zone for LS alignment, (**d**) plug flow zone for LS misalignment, (**e**) ideal mixing flow zone for LS alignment, (**f**) ideal mixing flow zone for LS misalignment.

All casting variants considered during the laboratory experiments were repeated six times. Based on the laboratory experiments, E-type residence time curves were recorded, on the basis of which the volumes of the stagnation, dispersed plug and ideal mixing zones were calculated. The results were subjected to statistical analysis, presenting the mean value (line crossing the box), the error range (box) and the standard deviation (segment lines). In the tundish, with a shallower immersion of the “umbrella” ladle shroud, the average stagnation flow value was 34.3% (Figure 2a). As the immersion of the ladle shroud increased, the stagnation zone decreased by 3.5%. In both cases, the side ports of the ladle shroud faced the outlets of the tundish. In the variant where the side ports of the ladle shroud faced the longitudinal side walls of the tundish, the average volume of the stagnation zone was 35% at both immersion depths (Figure 2a). When the side ports were located on the longitudinal walls of the tundish, the effect of the immersion depth of the ladle shroud on the volume of the stagnation zone, plug zone and ideal mixing zone disappeared (Figure 2a,c,e). However, the location of the side ports of the ladle shroud on the longitudinal walls of the tundish reduced the range of the dispersed plug flow zone to an average value of 4.5% (Figure 2c).

The highest dispersed plug flow volume value of 10.6% was recorded for casting variant 2 with deeper immersion of the ladle shroud and side ports directed towards the tundish outlets. In the case of the ideal mixing flow zone, its average value fluctuated around 60%, and only with a deeper immersion of the ladle shroud with the side ports directed towards the tundish outlets did the ideal mixing flow value decrease to 57.3% (Figure 2e). The experiments also tested the effect of the deviation of the ladle shroud from the vertical position. No significant effect of the ladle shroud deviation on the range of individual flow zones was observed (Figure 2b,d,f). Only in the case of a ladle shroud with side ports directed at the tundish outlets was a 3.2% increase in stagnant flow volume and a 1.9% decrease in dispersed plug flow volume observed in the case of deeper immersion. In the case of shallower immersion, the differences were close to 1%.

**Figure 3:**
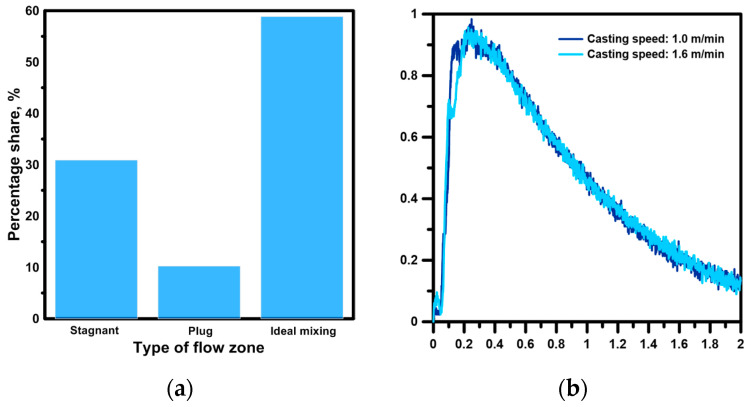
Hydrodynamics inside the tundish with a novel ladle shroud (case IX): (**a**) particular flow zones, (**b**) average residence time distribution curves.

An even more stable effect on the hydrodynamic structure in terms of the deviation of the ladle shroud from the vertical position was exerted by a ladle shroud with side ports directed towards the tundish longitudinal walls. In the tested range, the differences for the calculated flow zones did not exceed 1%. The results obtained for individual flow zones are characterized by a maximum error of 4%, with a fairly significant standard deviation, especially for casting variants in which the side ports were located on the tundish outlets. Therefore, with deeper immersion of the ladle shroud and the location of the side ports on the tundish outlets, increased dynamics of the supply stream’s impact on the formation of recirculation structures in the working volume of the tundish were observed. Figure 3b shows the average residence time curves for casting speeds of 1.0 m/min and 1.6 m/min. According to theory, an increase in momentum in a hydrodynamic system with the same reactor geometry should not significantly modify the flow paths. A comparison of the hydrodynamic system in the context of increased casting speed was performed for the optimal casting variant relating to the immersion of the ladle shroud and the location of the side ports (cases II and IX). The increase in casting speed did not significantly modify the hydrodynamic system in the working volume of the tundish in terms of the percentage share of individual flow types. The percentage changes in the volume of stagnant, dispersed plug and ideal mixing flows ranged from 1.5 to 0.4% (Figure 3a).

### 4.2. Numerical Model Validation

**Figure 4:**
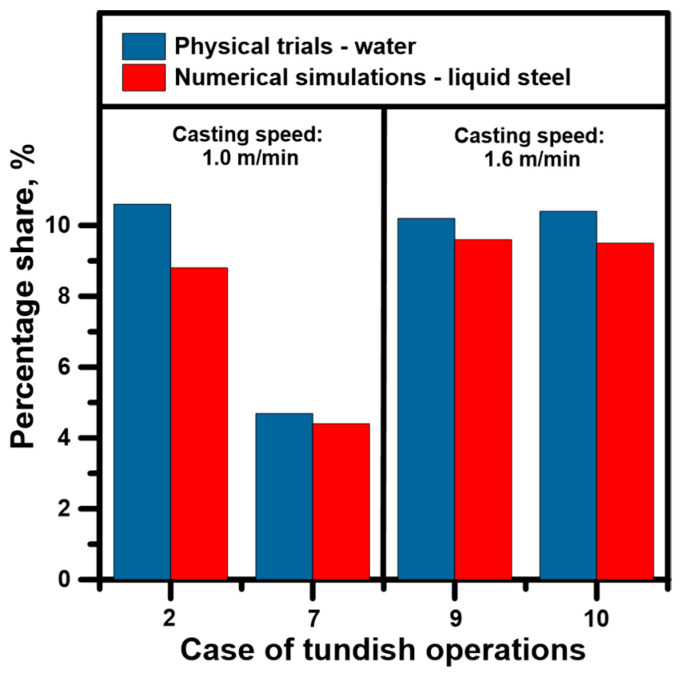
Dispersed plug flow zone calculated based on numerical modelling and physical trials.

A single-phase numerical model was selected for detailed diagnostics of the hydrodynamic structure forming in the tundish for specific casting conditions. Validations were performed for various casting cases, taking into account different immersion depths, the location of side ports and both casting speeds. Since the position of the point marking the appearance of the marker and the peak characterizing its maximum concentration precisely determine the percentage share of dispersed plug flow, plug flow was selected as the validation variable. Figure 4 shows the percentage share of dispersed plug flow volume. The differences between the average dispersed plug flow share calculated on the basis of experiments on a water model and calculated using a numerical model were 1.8%, 0.29%, 0.6% and 0.9% for casting variants 2, 7, 9 and 10, respectively. On this basis, it was concluded that the numerical model can correctly predict the flow of liquid steel and its interactions with the working space of the tundish.

### 4.3. Numerical Simulations—Influence of LS Type, LS Immersion Depth and Casting Speed on Hydrodynamics Inside the Tundish

**Figure 5:**
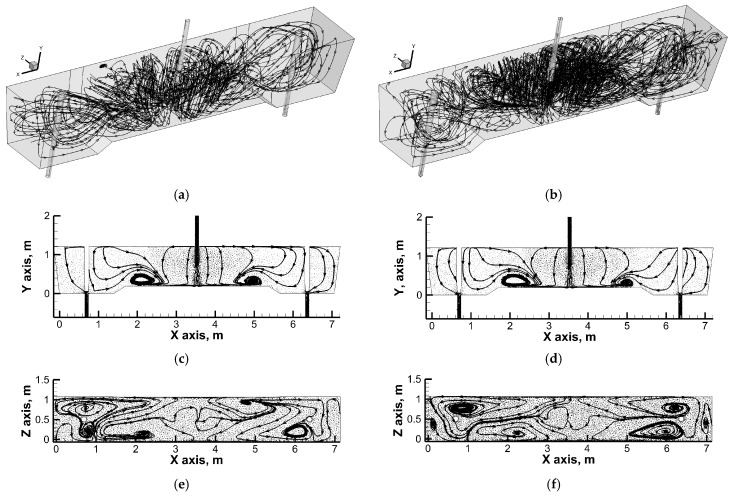
Liquid steel flow paths with directions (arrows): (**a**) global flow structure—SLS and casting speed: 1.0 m/min; (**b**) global flow structure—SLS and casting speed: 1.6 m/min; (**c**) flow at central vertical plane—SLS and casting speed: 1.0 m/min; (**d**) flow at central vertical plane—SLS and casting speed: 1.6 m/min; (**e**) flow at middle horizontal plane—SLS and casting speed: 1.0 m/min; (**f**) flow at middle horizontal plane—SLS and casting speed: 1.6 m/min.

**Figure 6:**
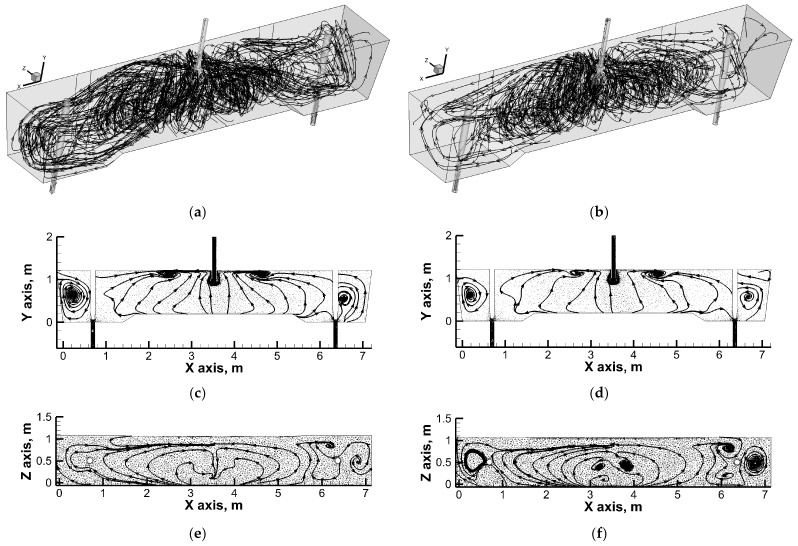
Liquid steel flow paths with directions (arrows): (**a**) global flow structure—ULS-O, normal immersion depth and casting speed: 1.0 m/min; (**b**) global flow structure—ULS-O, normal immersion depth and casting speed: 1.6 m/min; (**c**) flow at central vertical plane—ULS-O, normal immersion depth and casting speed: 1.0 m/min; (**d**) flow at central vertical plane—ULS-O, normal immersion depth and casting speed: 1.6 m/min; (**e**) flow at middle horizontal plane—ULS-O, normal immersion depth and casting speed: 1.0 m/min; (**f**) flow at middle horizontal plane—ULS-O, normal immersion depth and casting speed: 1.6 m/min.

**Figure 7:**
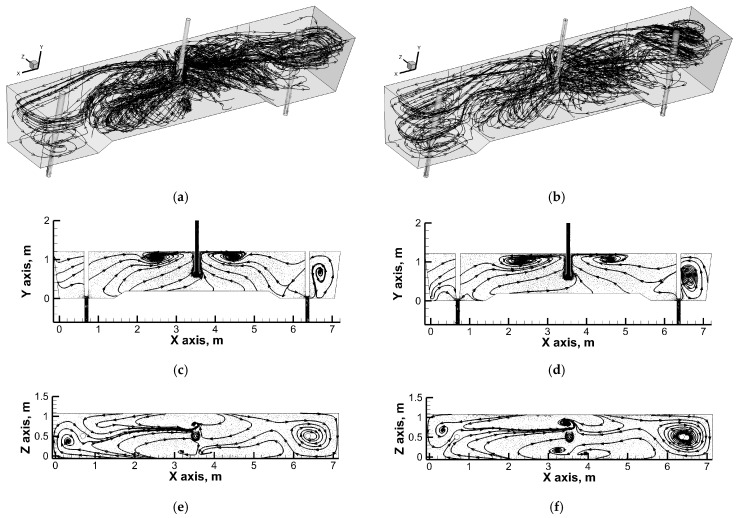
Liquid steel flow paths with directions (arrows): (**a**) global flow structure—ULS-O, deeper immersion depth and casting speed: 1.0 m/min; (**b**) global flow structure—ULS-O, deeper immersion depth and casting speed: 1.6 m/min; (**c**) flow at central vertical plane—ULS-O, deeper immersion depth and casting speed: 1.0 m/min; (**d**) flow at central vertical plane—ULS-O, deeper immersion depth and casting speed: 1.6 m/min; (**e**) flow at middle horizontal plane—ULS-O, deeper immersion depth and casting speed: 1.0 m/min; (**f**) flow at middle horizontal plane—ULS-O, deeper immersion depth and casting speed: 1.6 m/min.

Figure 5, Figure 6 and Figure 7 show the flow paths of molten steel in the working space of the tundish. In a tundish fed by a standard ladle shroud, distinct vertical recirculation structures are observed in the feed zone (Figure 5). Liquid steel streams flow from the longitudinal side walls towards the free surface and then descend towards the bottom in the central part of the tundish. In addition, in the central part of the tundish, reverse streams flow along the bottom from both stopper rod zones towards the feed zone. The vertical recirculation structures located in the feed zone partially evolve into ascending horizontal flow streams, which then descend towards the bottom just next to the transverse side walls of the tundish. An increase in casting speed does not significantly modify the hydrodynamic macro image. However, with a 60% increase in casting speed, the boundary between the vertical and horizontal recirculation zones is more pronounced. Feeding the tundish through an “umbrella” ladle shroud with side ports located on the tundish outlets significantly modifies the flow of liquid steel in the tundish (Figure 6). The direction of the flow of streams in the tundish feed zone changes. The liquid steel streams flow from the free surface towards the longitudinal side walls and then in the central part of the tundish as a result of mutual interaction, initiating upward movement.

**Figure 8:**
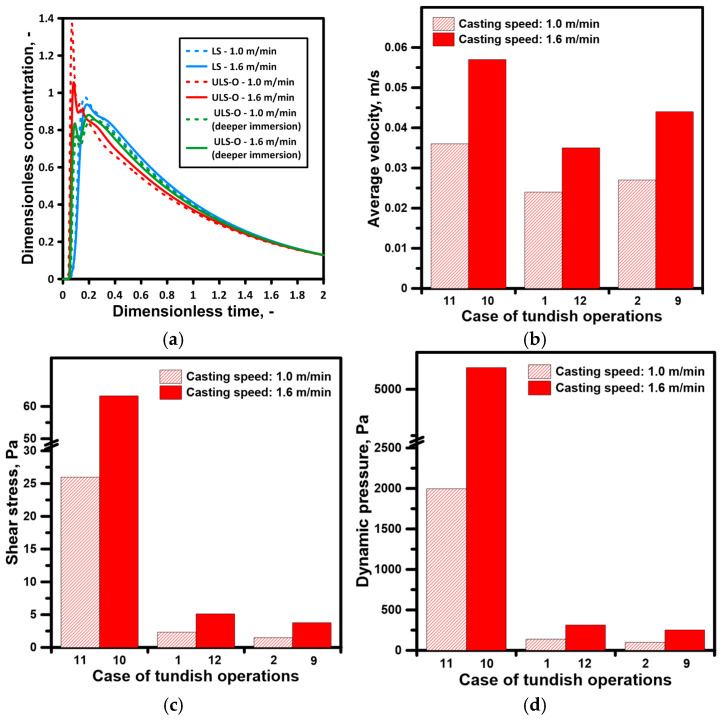
Influence of novel ladle shroud on liquid steel behaviour in the tundish: (**a**) residence time distribution curves, (**b**) average flow velocity, (**c**) shear stress in the tundish pouring zone, (**d**) dynamic pressure in the tundish pouring zone.

In addition, reverse streams flow towards the feed zone from the transverse side walls at the free surface. In contrast, the tundish outlet zones are partly fed by descending streams of stratified horizontal flow, which is clearly located at the longitudinal side walls of the tundish. An increase in casting speed increases the area of vertical recirculation to the lowered bottom zone. At the same time, part of the streams supplying the tundish pouring zones still flow to them along the longitudinal side walls. The most interesting hydrodynamic structure was observed when the immersion depth of the designed ladle shroud was increased (Figure 7). In the central part of the tundish, upward movement still dominates, although with a tendency towards horizontal flow coming from the stopper rod zones. In addition, horizontal streams supplying the tundish pouring zone, flowing from the free surface along the side walls, initiate horizontal recirculation in the stopper rod zone. The horizontal recirculation structure is maintained throughout the entire depth of the liquid steel along the stopper rods, regulating the flow rate of steel from the tundish to individual moulds. Figure 8a shows the residence time curves obtained from numerical simulations, on the basis of which the proportions of individual flow types were calculated.

In a tundish fed by a standard ladle shroud, the share of dispersed plug flow at a casting speed of 1.6 m/min was 9.5%. However, reducing the casting speed to 1.0 m/min resulted in a 1% decrease in the volume of dispersed plug flow. Hence, the residence time curves for the tundish fed by a standard ladle shroud further confirm the stability of hydrodynamic structures in the tested casting speed range. Furthermore, the numerically determined residence time curves also confirm a similar share of stagnant and active flows in the tundish with a standard ladle shroud and the “umbrella” ladle shroud tested at a deeper immersion and with side ports located on the tundish outlets (Figure 8a). In addition to determining hydrodynamic structures, the numerical model allows for the assessment of other important variables affecting the continuous casting process and the operation of the tundish. A 60% increase in casting speed resulted in an increase in the average velocity of liquid steel in the entire working volume of the tundish from 0.036 m/s to 0.057 m/s (tundish fed by an SLS). Average velocity was calculated by the numerical model based on fluid velocity in each cell of the analysed domain.

On the other hand, the use of the new proposed ladle shroud reduces the average velocity of liquid steel in the tundish from 0.024 m/s to 0.044 m/s, depending on the casting speed and the immersion depth of the ULS-O (Figure 8b). It means that the “umbrella” ladle shroud slows down the momentum of the stream flowing from the ladle, decreasing its impact on the liquid steel in the tundish. The reduction in flow velocity affects the distribution of shear stresses and dynamic pressure in the tundish feed zone, which is particularly exposed to the erosive effects of the feed stream. In the case of a standard ladle shroud, the shear stresses were 26 Pa and increased to 63 Pa with a 60% increase in casting speed (Figure 8c). However, using a unique ladle shroud reduced the shear stresses on the walls and bottom of the tundish in the feed zone to between 1.5 Pa and 5.1 Pa. An equally favourable result was obtained in the case of dynamic pressure, which in the case of a classic ladle shroud and a casting speed of 1.6 m/min was 5235 Pa and was reduced to 252 Pa (Figure 8d).

## 5. Summary

Based on laboratory investigations, including computer simulations and water model experiments, the effect of the influence of LS type, LS immersion depth, LS side ports position, LS misalignment and casting speed was examined. From the obtained results, it was found that:

1. For the “umbrella” ladle shroud, the optimal hydrodynamic structure (ratio of stagnant to active flow zones) was obtained for ULS with an immersion depth of 0.6725 m and with the side ports of ULS located on the tundish outlets.
2. With a three-degree deviation of the ULS from the vertical position, no significant changes in the hydrodynamic system were found in relation to the correctly mounted ladle shroud. From an industrial point of view, the absence of hydrodynamic stability loss due to incorrect installation of the ladle shroud is a significant advantage of the LS and its use under high-temperature conditions.
3. For both casting speeds considered, the use of the “umbrella” ladle shroud compared to the standard ladle shroud resulted in a reduction in the average steel flow velocity in the tundish in the range of 23–25% and 33–39% for deeper and shallower immersion, respectively.
4. The “umbrella” ladle shroud protects the refractory lining of the tundish in the tundish pouring zone, reducing tangential stresses and dynamic pressure on the tundish side walls and tundish bottom by 91–94% and 93–95%, respectively.

## Figures and Tables

**Table 1:** Considered cases of tundish operations with a novel ladle shroud.

Case No.	Type of LS	Casting Speed, m/min	LS Immersion Depth, m	LS Ports Side Position	LS Misalignment, °
1	ULS-O	1.0	0.36	outlets	0
2	ULS-O	1.0	0.6725	outlets	0
3	ULS-W	1.0	0.36	side walls	0
4	ULS-W	1.0	0.6725	side walls	0
5	ULS-O	1.0	0.36	outlets	3
6	ULS-O	1.0	0.6725	outlets	3
7	ULS-W	1.0	0.36	side walls	3
8	ULS-W	1.0	0.6725	side walls	3
9	ULS-O	1.6	0.6725	outlets	0
10	SLS	1.6	0.405	bottom	0
11	SLS	1.0	0.405	bottom	0
12	ULS-O	1.6	0.36	outlets	0

## Data Availability

The data presented in this study are available on request from the corresponding author due to legal restriction.
